# DLA class II risk haplotypes for autoimmune diseases in the bearded collie offer insight to autoimmunity signatures across dog breeds

**DOI:** 10.1186/s40575-019-0070-7

**Published:** 2019-02-15

**Authors:** Liza C. Gershony, Janelle M. Belanger, Andrea D. Short, Myly Le, Marjo K. Hytönen, Hannes Lohi, Thomas R. Famula, Lorna J. Kennedy, Anita M. Oberbauer

**Affiliations:** 1 Department of Animal Science, University of California, One Shields Ave, Davis, CA 95616 USA; 2 Brazilian National Council for Scientific and Technological Development (CNPq) fellow, Brasília, Brazil; 3 Centre for Integrated Genomic Medical Research, University of Manchester, Manchester, UK; 4 Research Programs Unit, Molecular Neurology, and Department of Veterinary Biosciences, University of Helsinki, Helsinki, Finland; 5 Folkhälsan Institute of Genetics, Helsinki, Finland

**Keywords:** (3–10): Dog, Autoimmune, MHC, DLA, Addison’s disease, SLO

## Abstract

**Background:**

Primary hypoadrenocorticism (Addison’s disease, AD) and symmetrical lupoid onychodystrophy (SLO) are two clinical conditions with an autoimmune etiology that occur in multiple dog breeds. In man, autoimmunity is associated with polymorphisms in immune-related genes that result in a reduced threshold for, or defective regulation of, T cell activation. The major histocompatibility complex (MHC) class II genes encode molecules that participate in these functions, and polymorphisms within these genes have been associated with autoimmune conditions in dogs and humans. Bearded collies have a relatively high prevalence of autoimmune diseases, particularly AD and SLO. Our study assessed the relationship between particular MHC (dog leukocyte antigen, DLA) class II haplotypes and the two autoimmune diseases most common in this breed. Moreover, five unrelated breeds at increased risk for AD were studied for comparative purposes and analyzed in the context of extant literature.

**Results:**

A single DLA class II three-locus haplotype, determined by sequence-based typing, was associated with increased risk for AD (DLA-DRB1*009:01/DQA1*001:01/DQB1*008:02) in bearded collies. Comparative analysis with the five additional breeds showed limited allele sharing, with DQA1*001:01 and DQB1*002:01 being the only alleles observed in all breeds. A distinct three-locus risk haplotype (DLA-DRB1*001:01/DQA1*001:01/DQB1*002:01) was associated with AD in the West Highland white terrier and Leonberger. Two different risk haplotypes were associated with increased risk for SLO in the bearded collie (DLA-DRB1*018:01/DQA1*001:01/DQB1*002:01 and DLA-DRB1*018:01/DQA1*001:01/ DQB1*008:02).

**Conclusion:**

Two-locus DQ haplotypes composed of DLA-DQA1*001:01 in association with DLA-DQB1*002:01 or DLA-DQB1*008:02 make up the four risk haplotypes identified in the present study and are also found in other risk haplotypes previously associated with diabetes mellitus and hypothyroidism across different dog breeds. Our findings build upon previously published data to suggest that this two-locus (DQ) model serves as a good indicator for susceptibility to multiple organ-specific autoimmune diseases in the canine population. However, it is also clear that additional loci are necessary for actual disease expression. Investigation of affected and unaffected dogs carrying these predisposing DQ haplotype signatures may allow for the identification of those additional genetic components that determine autoimmune disease expression and organ specificity.

**Electronic supplementary material:**

The online version of this article (10.1186/s40575-019-0070-7) contains supplementary material, which is available to authorized users.

## Plain english summary

Primary hypoadrenocorticism, also known as Addison’s disease (AD), and symmetrical lupoid onychodystrophy (SLO) are two autoimmune conditions that occur in multiple dog breeds. Disease expression depends on a combination of genetic and environmental factors, and discovery of genetic loci involved in disease susceptibility may help understand and predict risk for disease. Autoimmunity in humans is associated with altered immune-related genes that result in defective regulation of the immune system. The strongest associations for many human autoimmune diseases involve the major histocompatibility complex (MHC) class II genes. Genetic variants within these genes have also been associated with autoimmune conditions in dogs. Bearded collies have a relatively high prevalence of autoimmune diseases, and our study assessed the relationship between particular MHC (dog leukocyte antigen, DLA) class II haplotypes and the two autoimmune diseases most common in this breed, AD and SLO. We also studied five unrelated dog breeds at high risk for AD to determine if there were haplotypes common across affected dogs in these breeds and bearded collies with AD. A single DLA class II three-locus haplotype was associated with increased risk for AD in bearded collies with a different three-locus risk haplotype associated with AD in the West Highland white terrier and Leonberger. Two separate three-locus risk haplotypes were associated with increased risk for SLO in the bearded collie. Importantly, two-locus DQ haplotypes composed of DLA-DQA1*001:01 in association with DLA-DQB1*002:01 or DLA-DQB1*008:02 were common across the breeds and autoimmune conditions, and made up the four risk haplotypes identified in the present study. In the published literature, these same two-locus haplotypes are also found in risk haplotypes associated with diabetes mellitus and hypothyroidism across different dog breeds, suggesting that this two-locus model serves as a good indicator for susceptibility to multiple organ-specific autoimmune diseases in the dog population. However, many dogs carrying these haplotypes never develop clinical autoimmune disease, making it clear that additional genes are necessary for actual disease expression. Investigation of affected and unaffected dogs carrying these predisposing DQ haplotype signatures may allow for the identification of those additional genetic components that determine autoimmune disease expression and organ specificity.

## Background

Primary hypoadrenocorticism (Addison’s disease, AD) is a life-threatening clinical condition in dogs characterized by inadequate secretion of adrenocortical hormones by the adrenal glands [1–5]. In both humans and dogs, this endocrine disorder is caused by gradual immune-mediated destruction of the adrenal cortex [1, 4–7]. The presence of autoantibodies against adrenal antigens detected in both human [8, 9] and canine patients [2] with naturally occurring AD provide further evidence of the immune-mediated etiopathogenesis of AD. AD has been reported in many purebred and mixed breed dogs (OMIA 000519–9615) [1, 10, 11] with disease prevalence ranging from 0.06 to 0.4% in the overall dog population [12–15]. However, within certain breeds, prevalence of AD can be as high as 9% [7, 10, 16, 17]. Reported breeds at increased risk for developing AD include bearded collies, Portuguese water dogs (PWD), standard poodles, West Highland white terriers (WHWT), Leonbergers, Wheaten terriers and Nova Scotia duck tolling retrievers [1, 7, 10, 14, 16–20].

Symmetrical lupoid onychodystrophy (SLO) is another condition of autoimmune etiology that affects multiple dog breeds, such as the bearded collie, Gordon setter, English setter, giant schnauzer, Labrador retriever, Welsh corgi, boxer, and German shorthair pointer with variable prevalence (OMIA 001989–9615) [11, 21–25]. SLO is a clinical syndrome characterized by sloughing claws and secondary bacterial infection that was first described in 1992 [24, 26, 27]. Since then, most research has focused on disease diagnosis and treatment [22, 25]. While little research exists on the cause of SLO, Wilbe et al. [24] and Dahlgren et al. [28] have reported an association with particular major histocompatibility complex (MHC; or Dog Leukocyte Antigen, DLA) class II alleles in bearded collies, giant schnauzers, and English and Gordon setters [24, 28]. DLA class II haplotypes were also shown to be more prevalent among dogs of multiple breeds with AD [3].

Bearded collies, in particular, have a relatively high prevalence of autoimmune diseases. Health reports produced by the Bearded Collie Foundation for Health indicate 11.2% of registered dogs have one or more autoimmune disease [29]. Among these, more than half were diagnosed with AD or SLO. Autoimmune diseases such as AD and SLO are considered complex disorders that likely result from the combination of a predisposing genetic background and environmental factors [30]. The environmental triggers necessary or sufficient for autoimmune disease expression remain unclear, but discovery of the genetic loci involved in disease susceptibility may prove helpful in understanding and predicting risk for disease [31]. In humans, it is hypothesized that various polymorphisms in immune-related genes contribute to a reduced threshold for autoreactive lymphocyte activation and/or to defective regulation of autoreactive T cell responses [30]. Likely due to its role in recognition of self versus non-self, variation within MHC class II alleles has been implicated in multiple human autoimmune disorders including Addison’s disease, type 1 diabetes mellitus and inflammatory bowel disease [30, 32–34]. Similar connections have been made for dogs, and review of the published literature shows that several autoimmune conditions are associated with DLA class II risk haplotypes (as shown in Table 1) [3, 24, 28, 35–45].

**Table 1:** Published three-locus DLA class II haplotypes associated with increased risk for some immune-related diseases in dogs

Disease	DLA-DRB1	DLA-DQA1	DLA-DQB1	Breed(s)	Reference
AD	009:01	001:01	008:02	Bearded collie	Massey et al. 2013 [3]
	009:01	001:01	008:01:1	Cocker spaniel	
	015:01	006:01	023:01	Cocker spaniel, Springer spaniel	
	001:01	002:01	013:03	Springer spaniel	
	001:01	001:01	002:01	Labrador retriever, WHWT	
	015:02	006:01	023:01	Standard poodle	
	015:02	006:01	023:01	NSDTR	Hughes et al. 2010 [38]
SLO	018:01	001:01	008:02	Bearded collie, Gordon setter	Wilbe et al. 2010; Ziener et al. 2015 [24, 43]
	018:01	001:01	002:01	Bearded collie	Wilbe et al. 2010; Dahlgren et al. 2016 [24, 28]
	001:01	001:01	002:01	Giant schnauzer, English setter	
	101:02^a^	001:01	002:01	English setter	Dahlgren et al. 2016 [28]
Hypothyroidism	012:01	001:01	002:01	Doberman pinscher, Giant schnauzer	Kennedy et al. 2006; Wilbe et al. 2010 [40, 44]
	001:07	001:01	002:01	English setter	Ziener et al. 2015 [43]
	001:03	001:01	002:01	Gordon setter	
	001:01	001:01	002:01		
	049:01	010:01	019:01		
Diabetes mellitus	009	001	008	Multiple breeds	Kennedy et al. 2006 [39]
	015	006	023		
	002	009	001		
Hepatitis	006:01	004:01	013:03	Doberman pinscher	Dyggve et al. 2011 [36]
Anal furunculosis	001:01	001:01	002:01	German shepherd	Kennedy et al. 2008 [42]
Necrotizing meningoencephalitis	010:01:1	002:01	015:01	Pug	Greer et al. 2010 [37]
Meningoencephalitis	018:02	001:01	008:02	Greyhound	Shiel et al. 2014 [45]
	015:01	006:01	022:01		

*AD* Addison’s disease, *SLO* symmetrical lupoid onychodystrophy, *NSDTR* Nova Scotia duck tolling retriever, *WHWT* West Highland white terrier

^a^Current nomenclature is 001:07

Given the relatively high prevalence of autoimmune diseases in bearded collies, the present study assessed the relationship between particular MHC class II haplotypes and the two autoimmune conditions commonly observed in this breed. As a corollary, those haplotypes were evaluated in five other dog breeds at high risk for AD to determine if there were common DLA haplotype signatures associated with the disease across multiple breeds. This is the largest DLA study on AD and SLO bearded collies.

## Results

Two hundred and thirty-six bearded collies were haplotyped for the three polymorphic DLA class II genes: 125 healthy dogs (57 males, 68 females), 61 AD (22 males and 39 females) and 50 SLO (26 males, 24 females). Quality sequences of all three genes could not be obtained for three male control dogs, which were removed from further analysis.

Within the study population, six DLA-DRB1 alleles were identified, three of which were uncommon and only observed in healthy control dogs (allele frequency < 3%; Additional file 1: Table S1). Four DLA-DQA1 alleles were identified, two of which were only seen in a small number of controls (allele frequency < 3%). Similarly, seven DLA-DQB1 alleles were identified, three of which were less frequent (two found in control dogs only, and one in both AD and control dogs). Among the nine three-locus haplotypes identified (coded 1 through 9 for ease in presentation) in the bearded collie study population, four were infrequent in the breed (Table 2).

**Table 2:** Frequency, code, and odds ratio (OR) with 95% confidence interval (CI) of each three-locus haplotype observed in healthy, Addisonian (AD) and symmetrical lupoid onychodystrophy (SLO) bearded collies

Code	Haplotype	Controls		AD				SLO			
	DLA-DRB1/DQA1/DQB1	2*n* = 244	%	2*n* = 122	%	OR (95%CI)	*p*-value^†^	2*n* = 100	%	OR (95%CI)	p-value^†^
1	009:01/001:01/008:02	24	9.8	**25**	**20.5**	**2.36 (1.29–4.34)**	**0.0058**	1	1.0	**0.09 (0.01–0.70)**	**0.0047**
2	015:01/006:01/003:01	31	12.7	18	14.8	1.19 (0.64–2.22)	0.6263	4	4.0	**0.29 (0.10–0.83)**	**0.0172**
3	015:01/006:01/023:01	33	13.6	23	18.9	1.49 (0.83–2.66)	0.2177	2	2.0	**0.13 (0.03–0.55)**	**0.0013**
4	018:01/001:01/002:01	73	29.9	27	22.1	0.67 (0.40–1.11)	0.1355	**47**	**47.0**	**2.08 (1.29–3.35)**	**0.0029**
5	018:01/001:01/008:02	70	28.7	27	22.1	0.71 (0.42–1.18)	0.2094	**46**	**46.0**	**2.12 (1.31–3.43)**	**0.0026**
6	015:01/006:01/022:01	5	2.0	2	1.6	0.80 (0.15–4.17)	1	0	0.0	N/A	
7	002:01/009:01/001:01	6	2.5	0	0.0	N/A		0	0.0	N/A	
8	023:01/003:01/005:01	1	0.4	0	0.0	N/A		0	0.0	N/A	
9	015:02/006:01/023:01	1	0.4	0	0.0	N/A		0	0.0	N/A	

*N/A* not enough data points to calculate; ^†^Fisher’s exact *p*-value

Text in bold indicates the haplotype frequency significantly differed between cases and controls

### AD in bearded collies

Three DLA-DRB1 alleles (DLA-DRB1*009:01, DLA-DRB1*015:01 and DLA-DRB1*018:01) were frequent among bearded collie AD cases; DLA-DRB1*009:01 was the only allele associated with higher risk for AD (OR = 2.36, *p* = 0.0058; Additional file 2: Table S2). A single three-locus haplotype (haplotype 1), containing DLA-DRB1*009:01, was overrepresented among the AD dogs (Table 2). Increased risk for AD was observed in dogs that carried haplotype 1 although only two AD cases were homozygous for this haplotype. The majority of AD cases were heterozygous for haplotype 1 (*n* = 21), with half of those (*n* = 11) also carrying haplotype 5. Bearded collies carrying the heterozygous genotype 1 5 were at higher risk for AD (Table 3). No differences in DLA homozygosity were noted when comparing AD dogs to controls (Table 4).

**Table 3:** DLA class II genotypes significantly associated with Addison’s disease (AD) and symmetrical lupoid onychodystrophy (SLO) in 233 Bearded collies, and corresponding odds ratios (OR) with the 95% confidence interval (CI) using the DLA haplotype codes reported in Table 2

Disease	Genotypes	Controls		Cases		p-value^†^	OR (95% CI)
		*n*	%	*n*	%		
AD	All genotypes containing haplotype 1	23	18.9	23	37.7	0.0070	2.6 (1.31–5.19)
AD	Genotype 1 5	7	5.7	11	18.0	0.0154	3.6 (1.32–9.87)
SLO	All genotypes containing haplotype 4	62	50.8	36	72.0	0.0115	2.5 (1.22–5.07)
SLO	All genotypes containing haplotype 5	59	48.4	35	70.0	0.0114	2.5 (1.24–5.02)
SLO	Genotype 4 4	11	9.0	11	22.0	0.0258	2.8 (1.14–7.08)
SLO	Genotype 5 5	11	9.0	11	22.0	0.0258	2.8 (1.14–7.08)
SLO	Genotype 4 5	23	18.9	22	44.0	0.0011	3.4 (1.65–6.94)
SLO	Genotypes containing haplotypes 4 and/or 5	98	80.3	49	98.0	0.0033	12.0 (1.58–91.33)

^†^Fisher’s exact *p*-value, significant at *p* < 0.05

**Table 4:** Homozygosity across DLA class II genes in 233 Bearded collies (61 AD, 50 SLO and 122 controls). Different letters in the same column indicate statistical difference (*p* < 0.05) in DLA homozygosity across health status

	DLA-DRB1		DLA-DQA1		DLA-DQB1		DLA-DRB1/DQA1/DQB1	
	*n*	%	*n*	%	*n*	%	*n*	%
AD	26	42.6%^a^	45	73.8%^a^	23	37.7%^a^	12	19.7%^a^
SLO	45	90.0%^b^	46	92.0%^b^	23	46.0%^a^	22	44.0%^b^
Controls	62	50.8%^a^	79	64.8%^a^	36	29.5%^a^	29	23.8%^a^

*n* = number of homozygous dogs

*AD* Addison’s disease, *SLO* symmetrical lupoid onychodystrophy

**Fig. 1:**
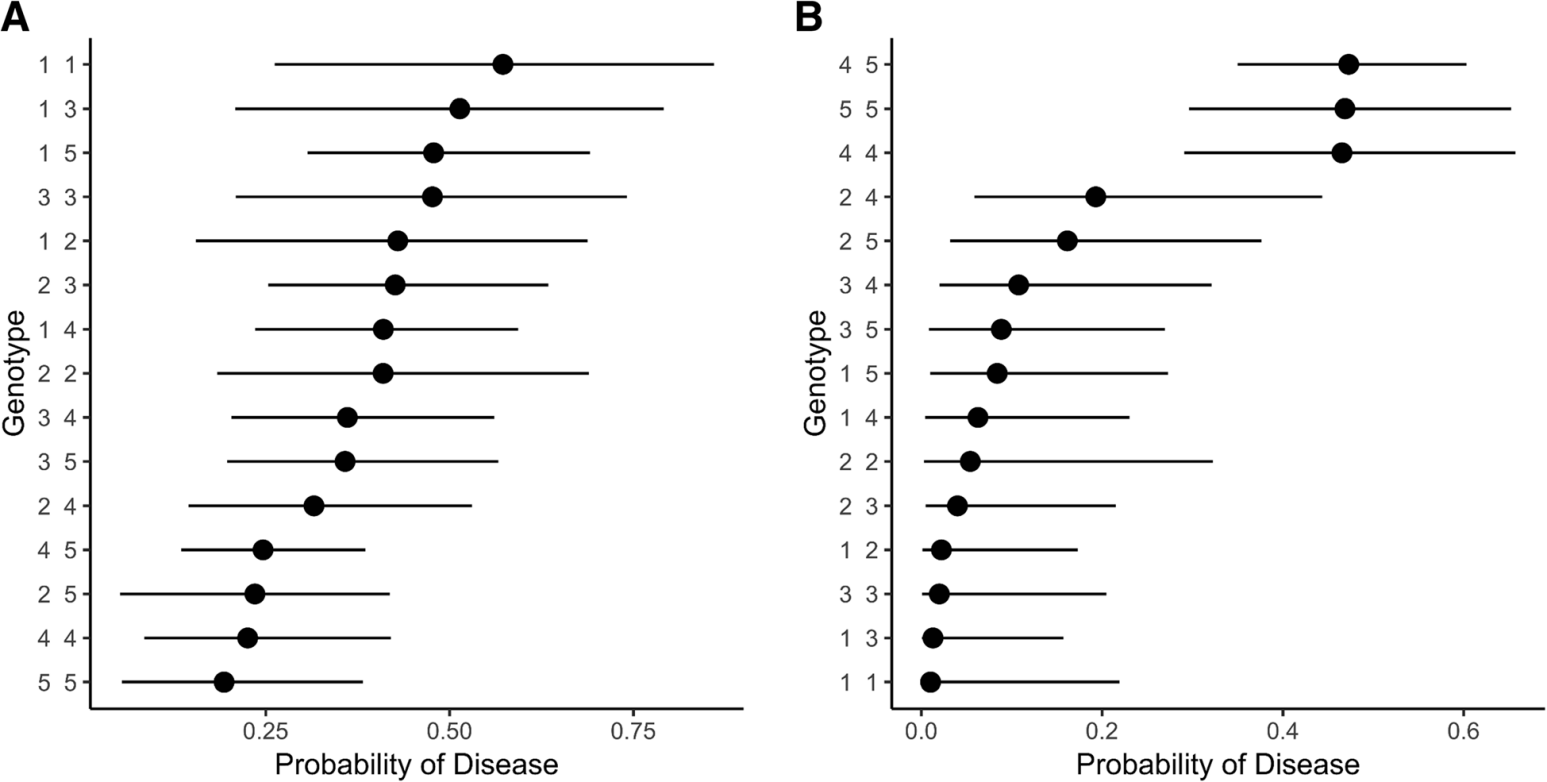
Probability of (**a**) Addison’s disease and (**b**) symmetrical lupoid onychodystrophy associated with the most common genotypic combinations of three-locus haplotypes observed in bearded collies (*n* = 219). Dots indicate the estimated probability of disease and lines represent the 95% confidence interval. DLA haplotype codes are as reported in Table 2

A subset of bearded collies (*n* = 219) that excluded those dogs carrying less frequent haplotypes (i.e., haplotypes 6–9; *n* = 14) was subjected to pairwise genotypic comparisons using logistic regression. A comparison between healthy and AD dogs showed genotypes 4 4, 4 5, and 5 5 were associated with the lowest probability for AD and genotypes that included haplotype 1 were associated with the highest probability (Fig. 1a). No sex differences were observed in the analysis (*p* > 0.05).

A second data set containing a total of 75 United Kingdom bearded collies (29 AD, 46 controls) included in a previously published study [3] was analyzed for comparison. Common haplotypes in the bearded collie breed (haplotypes 1–5) were seen in similar proportions in both data sets, and analyzing the merged data further corroborated the association between haplotype 1 and AD (OR = 2.87; 95% CI = 1.76–4.66; *p* = 0.00002; Additional file 3: Table S3). Six three-locus haplotypes observed in the published data (allele frequency < 3%) were not seen in the new dataset. Conversely, haplotype 6 from the new dataset had not been observed in the published data. The combined dataset showed haplotype 4 underrepresented in AD dogs. To account for possible location influences, a geographical analysis of haplotype frequency in the combined dataset, which consisted predominantly of dogs from North America and Europe, showed differences in haplotype frequency between North American and European bearded collies (Additional file 4: Table S4). Haplotype 3 was more prevalent among North American control and AD dogs, whereas haplotype 4 was more prevalent among European controls and haplotype 2 was more prevalent in North American AD dogs. Nevertheless, haplotype 1 remained associated with increased risk for AD in both regions (Additional file 5: Table S5). No significant differences were seen in haplotype frequency between the regions for SLO dogs. The combined dataset also included dogs from Australia (7 controls and 1 SLO) and New Zealand (2 controls, 2 AD and 1 SLO), however the low sample numbers precluded meaningful statistical comparisons for those regions.

### AD in other breeds

Seventeen DLA-DRB1 alleles were identified among all the breeds assessed, including the bearded collies (Additional file 6: Table S6). Seven of those were shared by three or more breeds whereas five were seen in only one of the breeds. The DLA-DRB1*008:02 and -DRB1*011:01 alleles were only seen in the PWD, DLA-DRB1*016:01 in the Leonberger, DLA-DRB1*017:01 in the WHWT and DLA-DRB1*084:01 in the Labradoodle. Among the seven DQA1 alleles, four were shared by three or more breeds. DLA-DQB1 was diverse across breeds, with 16 different alleles noted, only five of which were seen in more than two breeds. Despite the considerable diversity of alleles seen among the three polymorphic DLA class II genes across the six studied breeds at increased risk for AD, DLA-DQA1*001:01 and DQB1*002:01 were the only alleles shared by all six breeds.

Individual DLA class II alleles were significantly associated with AD in the WHWT (DLA-DQA1*001:01 and DLA-DQB1*002:01 - Additional file 7: Table S7) and Leonbergers (DLA-DRB1*001:01 - Additional file 8: Table S8). These alleles are a part of a three-locus haplotype that was significantly associated with AD status in WHWTs and Leonbergers (haplotype 14; Table 5). No alleles showed association with AD in the PWDs (Additional file 9: Table S9), standard poodles (Additional file 10: Table S10) and Labradoodles (Additional file 11: Table S11). Twenty-nine three-locus haplotypes were identified across the six studied breeds, five of which were shared by more than three breeds (Table 5 - haplotypes 3, 9, 12, 13 and 14). Haplotype 14 was the only one associated with risk for AD in our studied breeds. We confirm its association with AD in the WHWT, as previously reported [3], and identify it as the first risk haplotype associated with AD in the Leonberger. No association was seen for the three other breeds that carried haplotype 14 (Labradoodle, PWD and standard poodle).

**Table 5:** Frequency, code and odds ratio (OR) for DLA class II three-locus haplotypes observed in the six studied breeds at increased risk for Addison’s disease (AD): bearded collie (61 cases, 122 controls), West Highland white terrier (WHWT; 43 cases, 166 controls), standard poodles (30 cases, 55 controls), Portuguese water dog (PWD; 17 cases, 76 controls), Labradoodle (12 cases, 9 controls) and Leonberger (11 cases, 14 controls)

Code	DRB1	DQA1	DQB1	Breed	Control (2*n*)	AD (2*n*)	OR	95% CI	*p*-value^†^
1	009:01	001:01	008:02	**Bearded collie**	**24**	**25**	**2.36**	**1.29–4.34**	**0.0058**
2	015:01	006:01	003:01	Bearded collie	31	18	1.19	0.64–2.22	0.6263
3	015:01	006:01	023:01	Bearded collie	33	23	1.49	0.83–2.66	0.2177
				Labradoodle	2	8	4.0	0.73–21.8	0.1466
				Standard poodle	68	37	0.99	0.52–1.90	1
				WHWT	17	0	N/A		
4	018:01	001:01	002:01	Bearded collie	73	27	0.67	0.40–1.11	0.1355
				WHWT	1	0	N/A		
5	018:01	001:01	008:02	Bearded collie	70	27	0.71	0.42–1.18	0.2094
6	015:01	006:01	022:01	Bearded collie	5	2	0.80	0.15–4.17	1
				Labradoodle	0	1	N/A		
7	002:01	009:01	001:01	Bearded collie	6	0	N/A		
				Labradoodle	0	1	N/A		
				Standard poodle	2	0	N/A		
8	023:01	003:01	005:01	Bearded Collie	1	0	N/A		
				PWD	11	5	2.21	0.71–6.84	0.1775
9	015:02	006:01	023:01	Bearded collie	1	0	N/A		
				Labradoodle	4	3	0.50	0.10–2.58	0.6786
				Standard poodle	7	8	2.26	0.78–6.59	0.1586
				PWD	11	5	2.21	0.71–6.84	0.1775
				WHWT	4	0	N/A		
10	015:01	006:01	020:02	WHWT	51	6	0.41	0.17–1.00	0.0514
11	013:01	001:01	002:01	Leonberger	4	4	1.22	0.27–5.59	1
				Standard poodle	1	0	N/A		
				WHWT	1	0	N/A		
12	006:01	005:01:1	007:01	Labradoodle	1	2	1.55	0.13–18.5	1
				Leonberger	5	1	0.2	0.02–1.86	0.1986
				Standard poodle	4	0	N/A		
				WHWT	2	0	N/A		
13	009:01	001:01	008:01:1	Labradoodle	1	3	2.43	0.23–25.5	0.6227
				Standard poodle	5	2	0.72	0.14–3.85	1
				WHWT	3	2	2.61	0.43–15.88	0.5882
				PWD	8	0	N/A		
14	001:01	001:01	002:01	Labradoodle	2	2	0.73	0.09–5.72	1
				**Leonberger**	**11**	**16**	**3.64**	**1.07–12.30**	**0.0447**
				PWD	57	15	1.32	0.62–2.79	0.5597
				**WHWT**	**130**	**51**	**2.3**	**1.40–3.67**	**0.0009**
				Standard poodle	3	0	N/A		
15	001:01	001:01	036:01	Standard poodle	2	0	N/A		
				WHWT	6	0	N/A		
				Labradoodle	0	1	N/A		
16	001:01	009:01	001:01	WHWT	77	14	0.64	0.34–1.21	0.1886
17	008:02	003:01	004:01	PWD	59	9	0.57	0.25–1.30	0.2373
18	011:01	002:01	013:03	PWD	2	0	N/A		
19	012:01	004:01	013:03	PWD	3	0	N/A		
20	012:01	004:01	013–017	Standard poodle	4	0	N/A		
21	015:01	006:01	011:01	WHWT	25	5	0.76	0.28–2.04	0.6499
22	015:01	006:01	026:01	Labradoodle	1	2	1.55	0.13–18.5	1
23	015:01	009:01	001:01	Labradoodle	3	1	0.22	0.02–2.29	0.2972
				Standard poodle	8	9	2.25	0.82–6.18	0.1792
				WHWT	14	6	1.70	0.63–4.57	0.3926
24	015:02	006:01	026:01	Standard poodle	0	1	N/A		
25	015:03	006:01	023:01	Labradoodle	2	0	N/A		
				Standard poodle	4	2	0.91	0.16–5.14	1
26	016:01	001:01	002:01	Leonberger	6	1	0.16	0.02–1.44	0.1064
27	017:01	002:01	013:03	WHWT	1	0	N/A		
28	020:01	004:01	013:03	PWD	1	0	N/A		
				Standard poodle	2	1	0.92	0.08–10.3	1
29	084:01	009:01	001:01	Labradoodle	2	0	N/A		

*CI* confidence interval, *N/A* not enough data points to calculate; ^†^Fisher’s exact p-value, significant at *p* < 0.05

N.B. DLA-DQB1*013–017 is short hand for DQB1*01303 and 01701 appearing on the same haplotype

Bolded values were statistically significant at α = 0.05. Haplotype codes 1–9 are as used in the text, additional codes were added as needed

### SLO in bearded collies

Whereas DLA-DRB1*018:01 and -DQA1*001:01 were common alleles in the bearded collie, they were present at a much higher frequency among SLO dogs compared to healthy controls with OR = 9.38 (*p* = 7.40 × 10^− 10^) and OR = 7.22 (*p* = 4.19 × 10^− 7^), respectively, for SLO disease risk (Additional file 12: Table S12). The majority of SLO dogs (49/50) carried a DLA-DQB1*002:01 and/or DLA-DQB1*008:02 allele although an increased risk for disease was observed only for DLA-DQB1*002:01 (OR = 2.08, *p* = 0.0029). However, both DQB1 alleles were associated with DLA-DRB1*018:01, and the three-locus haplotypes containing these combinations (haplotypes 4 and 5) were significantly overrepresented among SLO dogs and associated with increased risk for disease (Table 2). While risk for disease was slightly higher for the heterozygous genotype 4 5, dogs homozygous for each of the risk haplotypes showed similar risk for disease (Table 3). Despite this, SLO dogs were significantly more homozygous in their DLA-DRB1 (OR = 8.71; 95% CI = 3.24–23.43; *p* = 1.85 × 10^− 6^) and -DQA1 (OR = 6.26; 95% CI = 2.11–18.56; *p* = 0.0003) genes than controls (Table 4). Homozygosity across all three loci was also significantly greater among SLO dogs compared to controls (OR = 2.52; 95% CI = 1.26–5.06; *p* = 0.0104).

Pairwise genotypic comparisons using logistic regression revealed two major genotype groupings, with genotypes 4 4, 4 5 and 5 5 significantly associated with a higher probability for SLO when compared to genotypes 1 4, 2 3, 1 2, 3 3, 1 3 and 1 1 (Fig. 1b); the inverse of what was seen for AD. Genotypes composed of haplotypes 1 and/or 2 were associated with a reduced probability of having SLO. No sex differences were observed in the analysis (*p* > 0.05). When geographical regions were considered in the analysis, haplotype 4 remained a risk haplotype for SLO in Europe, but not in North America (Additional file 13: Table S13). Conversely, haplotype 5 remained a risk for SLO in North America, but not in Europe.

## Discussion

Some of the strongest genetic associations with human autoimmune diseases, such as AD and type 1 diabetes mellitus, involve MHC class II genes [46]. The present study identified a single DLA class II risk haplotype for AD in the bearded collie, consistent with a previous report in a smaller number of dogs from the United Kingdom [3], although our data did not support the researchers’ observation of haplotype 4 being protective for AD. While highly prevalent among control dogs, this haplotype was also observed at similar proportions in AD bearded collies from our dataset. However, when data from the previously published paper was merged with the newly acquired data, a combined analysis showed that haplotype 4 became slightly underrepresented among cases. This may be due to differences in the geographic origin of the samples. Haplotype 4 was more prevalent among controls of European origin and a risk haplotype for SLO in European bearded collies, but not among those from North America. Conversely, haplotype 5 remained a risk for SLO in dogs sampled from North America, but not those from Europe. This finding could indicate actual geographical differences in susceptibility to disease, although it is more likely that they reflect the significantly reduced sample sizes for SLO cases when the data was split by geographical region, thus reducing our power to detect true associations. As for AD, regional differences in haplotype frequency did not affect our initial observation, and haplotype 1 remained significantly associated with AD in both Europe and North America.

Surprisingly, the highest risk for AD was seen when haplotype 1 was combined with the SLO risk haplotype 5. The sole difference between these two haplotypes is the DLA-DRB1 allele: DLA-DRB1*009:01 and DLA-DRB1*018:01, respectively. The non-polymorphic DLA-DRA and polymorphic -DRB1 genes encode two chains (alpha and beta, respectively) of the same MHC class II molecule, whereas the DLA-DQA1 and -DQB1 genes encode chains that combine to form a different MHC class II molecule [47]. MHC molecules actively participate in the positive and negative selection of developing T cells in the thymus. The recognition of self-peptides associated with MHC molecules results in selection of T cells that bind MHC with intermediate-to-low avidity; binding that is too strong represents potential for autoreactivity and results in deletion of such T cells. Moreover, intermediate avidity, where binding is strong but falls below the threshold for deletion, results in generation of regulatory T cells capable of suppressing self-antigen presentation in the peripheral lymph nodes, thus preventing autoreactivity [46]. While the exact mechanism through which MHC genes contribute to autoimmune disease development is not fully understood, disease-predisposing MHC molecules appear to confer risk by allowing autoreactive T cells to escape central tolerance, whereas protective MHC molecules confer resistance to autoimmunity by promoting negative selection and generation of regulatory T cells [46, 47]. The two DLA-DRB1 alleles mentioned above produce molecules that differ by four amino acids in the hypervariable regions of the gene, which encode the peptide-binding region of an MHC molecule. Amino acid changes in the peptide-binding region of MHC molecules can affect the repertoire of peptides they are capable of presenting to T cells [46], which is why MHC heterozygosity is generally associated with increased fitness due to the ability to detect a larger number of pathogenic antigens compared to a homozygous individual [46, 48]. However, carrying two autoimmune disease-predisposing MHC haplotypes may actually increase the number and types of autoreactive T cells that escape central tolerance thus increasing the risk of developing autoimmunity. In fact, humans who are heterozygous for the MHC class II risk haplotypes, HLA-DR3 and HLA-DR4, are at much higher risk for autoimmune type 1 diabetes and AD than those with either of the homozygous haplotypes [32, 33, 49]. This may also be the case for bearded collies with AD, where most dogs were heterozygous for the risk haplotype and increased risk was seen in association with the heterozygous 1 5 genotype.

As expected, considerable diversity of alleles was seen across the six dog breeds at increased risk for AD, but allele sharing was fairly limited. Five of the 29 three-locus haplotypes identified were shared by more than three of the studied breeds (haplotypes 3, 9, 12, 13 and 14), four of which had been previously associated with AD in the cocker spaniel, springer spaniel, WHWT, Labrador retriever, standard poodle and Nova Scotia duck tolling retriever [3, 38]. However, our study only confirmed association of haplotype 14 with AD in the WHWT and also identified it as the first risk haplotype associated with AD in the Leonberger. Haplotype 9, previously associated with AD in the standard poodle [3], was not confirmed as a risk haplotype for AD in our analysis. Although our dataset had more AD standard poodles, our control group is smaller than the previous study, which may have impaired our ability to detect the association. The absence of an association may also be due to differences between studied populations: our dataset consisted of standard poodles from the United States and United Kingdom, whereas previously published data consisted of dogs almost exclusively from the United Kingdom.

Two risk haplotypes were identified for SLO in our bearded collies, consistent with previous findings [24] though that study evaluated only Scandinavian bearded collies using many fewer dogs. Furthermore, in our population, almost half of the SLO dogs were homozygous for one of these two risk haplotypes (i.e. genotypes 4 4 or 5 5). Interestingly, although the highest risk was seen with the heterozygous genotype 4 5, dogs homozygous for either of the risk haplotypes appear to be at similar risk for disease. This may be explained by the high similarity between haplotypes 4 and 5, which differ only in their DLA-DQB1 allele. DLA-DQB1*002:01 (in haplotype 4) differs from DLA-DQB1*008:02 (in haplotype 5) by three neighboring amino acids, only one of which falls within a hypervariable region in exon 2, as defined by Kennedy et al. in 1999. Since the alleles have similar hypervariable regions, they likely have similar antigen binding and functional properties [50], which could explain why genotypes 4 4, 4 5, and 5 5 all offered similar risk for SLO.

Three three-locus haplotypes were associated with reduced risk for expressing SLO: haplotypes 1, 2 and 3. Despite its association with increased risk for AD, haplotype 1 was associated with a lower risk of expressing SLO. In fact, as highlighted above, the sole difference between the SLO risk haplotype 5 and the AD risk haplotype 1 is the DRB1 allele, which may suggest a role for this gene in determining the target tissue for autoimmune disease in the bearded collie. However, strong linkage disequilibrium (LD; non-random association between alleles) in the region may actually be responsible for this observation, and it is possible that tissue-specific genetic determinants exist in nearby genes that are in strong LD with the DLA-DRB1 gene resulting in the observed association.

Perhaps most interesting, however, is that many of the risk haplotypes associated to date with organ-specific autoimmune diseases in dogs carry one of two DQ haplotypes: DLA-DQA1*001:01/DQB1*002:01 (DQ1) or DLA-DQA1*001:01/DQB1*008:02 (DQ2). The DQ1 haplotype is part of haplotype 14, for instance, which has not only been associated with AD in WHWT and Leonbergers, but also with anal furunculosis in the German shepherd [42], hypothyroidism in the Gordon setter [43] and SLO in the giant schnauzer and English setter [24]. Furthermore, the DQ1 combination is found in two different three-locus haplotypes that have been associated with higher risk for hypothyroidism (lymphocytic thyroiditis) in Doberman pinschers, giant schnauzers (DLA-DRB1*012:01/DQA1*001:01/DQB1*002:01) and English setters (DLA-DRB1*001:07/DQA1*001:01/DQB1*002:01) [40, 43, 44]. Moreover, the four three-locus DLA class II risk haplotypes associated with SLO in different dog breeds [24, 28, 43] carry a DQ1 or DQ2 haplotype. These DQ haplotypes were also observed in two SLO-affected dogs in our database that represent breeds in which SLO is uncommon: one great dane, heterozygous for DQ1, and one Belgian tervuren, homozygous for DQ2 (unpublished finding). DQ2 is found in haplotype 1, the risk haplotype for AD in the bearded collie and diabetes mellitus in multiple dog breeds [39]. It is also a part of haplotype 5, which is associated with risk for SLO in the bearded collie and Gordon setter [24, 28, 43], and a three-locus haplotype associated with meningoencephalitis in the greyhound (DLA-DRB1*018:02/DQA1*001:01/DQB1*008:02) [45]. These observations indicate that DQ haplotypes may constitute signatures of autoimmune predisposition across multiple dog breeds, which perhaps combine with breed-specific genetic components to determine the tissue-specificity of autoimmune disease expression.

Whereas an association between MHC class II haplotypes, particularly DQ haplotypes, and autoimmune disease clearly exists in both dogs and humans, other MHC or non-MHC genetic components likely play a role in autoimmune disease development, which would explain why many dogs carrying the risk haplotype for a disease, such as AD, SLO or anal furunculosis, fail to develop the condition. Although our study points to a strong influence of particular DQ haplotypes in different autoimmune diseases across multiple dog breeds, the fact that a risk haplotype can be associated with a disease in one dog and not another suggests that other loci are necessary to determine disease expression and target organ specificity. In fact, studies in mice have shown that, while MHC genes play a major role in autoimmunity, they are still only a part of the genetic components required for disease development. Studies using nonobese diabetic (NOD) mice, which carry a particular MHC haplotype and are prone to developing diabetes, show that the presence of the diabetes-prone MHC haplotype in a different mouse strain fails to cause diabetes [51]. In contrast, when the diabetes-prone MHC of NOD mice is replaced by a different haplotype, autoimmunity develops but in a different target organ [52], thereby demonstrating tissue-specific susceptibility dependent upon factors additional to the MHC. Therefore, autoimmune disease expression is likely dependent upon the existence of non-MHC genetic determinants cooperating with autoimmune disease-predisposing MHC class II haplotypes.

## Conclusion

Our study results complement published data, and shows that three DLA class II risk haplotypes associated with autoimmune diseases are highly prevalent in the bearded collie population, which may account for incidence of autoimmune disorders in the breed. Moreover, the two-locus DQ haplotypes making up these three risk haplotypes are found in other risk haplotypes associated with diabetes mellitus and hypothyroidism across different dog breeds. Multiple studies have clearly demonstrated that DLA class II genes play a role in canine autoimmunity, although associations have been deemed breed- and disease-specific. Our work, in combination with the published literature, revealed common DLA-DQ haplotypes associated with different autoimmune diseases across multiple dog breeds. While additional loci are clearly necessary for actual disease expression, the DQ two-locus model may be a good indicator for susceptibility to certain organ-specific autoimmune diseases in dogs, and future studies on carriers of these risk DQ haplotypes may prove fruitful in identifying the additional genetic components involved in canine autoimmunity. These markers may then be used to make informed breeding decisions with the purpose of reducing the incidence of each disease in the canine population. Furthermore, genetic markers of canine autoimmune diseases may provide insights into their human counterpart.

## Methods

### Samples

Blood or buccal swab samples were obtained from 236 bearded collies (125 healthy, 61 AD and 50 SLO) in North America, Europe, Australia and New Zealand that were healthy or affected by one of two autoimmune diseases (AD or SLO). Addisonian cases consisted of dogs diagnosed by a veterinarian on the basis of clinical signs, demonstrated electrolyte imbalance (sodium to potassium ratio < 27:1) and confirmed with an ACTH stimulation test. Serum cortisol levels < 2.0 μg/dL (55 nmol/L) before and after ACTH administration were considered diagnostic of AD. Dogs with a history of corticosteroid use that may have interfered with the ACTH stimulation test were excluded, as well as dogs presenting with atypical AD, characterized by low levels of serum cortisol before and after ACTH administration, but normal electrolyte ratio [19]. SLO cases consisted of dogs diagnosed by a veterinarian through biopsy and/or clinical findings such as pain, abnormal growth of nails, or nails bleeding, splitting or falling off. Control dogs were nine years or older, with no history of autoimmune disease (suspected or diagnosed) and considered healthy based on medical history. Samples were subjected to DNA extraction as previously described [53] and quantified using a Nanodrop® spectrophotometer. DNA samples were stored at − 20 °C until processing. The same procedure was performed for five other dog breeds at increased risk for developing AD: standard poodles (11 cases, 10 controls), WHWT (10 cases, 10 controls), Labradoodles (9 cases, 9 controls), PWD (15 cases, 16 controls) and Leonbergers (11 cases, 13 controls).

To complement the AD data, DLA haplotypes from a previously studied population [3] were included in the analyses for the bearded collie (29 cases, 46 controls from the UK for a total of 90 cases and 168 controls), standard poodle (19 cases, 45 controls from the UK and North America for a total of 30 cases and 55 controls), WHWT (33 cases, 156 controls from the UK for a total of 43 cases and 166 controls), Labradoodle (3 cases from the UK for a total of 12 cases and 9 controls), and PWD (2 cases, 60 controls from the USA and UK for a total of 17 cases and 76 controls). The z-ratio test for independent proportions (http://vassarstats.net/propdiff_ind.html) was used to assess differences between geographical regions in the combined dataset [54].

### DLA Haplotyping

Sequence based typing was used to determine DLA haplotypes of all dogs. Amplification and sequencing of exon 2 for each of the three polymorphic MHC class II genes, DLA-DRB1, −DQA1 and -DQB1, were performed using flanking primers as previously described [55–57] and two new primer sets developed for DLA-DRB1 and DQB1 (Additional file 14: Table S14). Sequences from the newly designed primers provided greater coverage upstream and downstream of exon 2, improving sequence quality obtained for the entire exon, and matched bearded collie DQB1 (*n* = 30) and DRB1 (*n* = 18) sequences obtained using published primers. A touchdown polymerase chain reaction (PCR) protocol was initially used for all three genes, which consisted of 14 touchdown cycles with annealing temperatures starting at 62 °C for DLA-DRB1, 57 °C for DLA-DQA1 and 73 °C for DLA-DQB1, and reducing by 0.5 °C each cycle, and 24 additional cycles with annealing temperatures of 55 °C for DLA-DRB1, 50 °C for DLA-DQA1 and 66 °C for DLA-DQB1. The same touchdown PCR protocol was used with the newly designed DLA-DQB1 primer set, but a standard PCR protocol (95 °C denaturation, 65 °C annealing, 72 °C extension, 30 cycles) was used with the new set of DLA-DRB1 primers. Promega GoTaq® Flexi DNA Polymerase (Promega, WI, USA) was used for all PCRs in a 25 μL reaction. Size and concentration of amplicons were verified by running 5 μL of the PCR product on a 1% agarose gel. PCR products were then purified using Exosap-IT™ (Thermo Fisher Scientific, Waltham, MA, USA) according to manufacturer’s recommendations, and sequenced by capillary electrophoresis on an Applied Biosystems 3730 (UC Davis DNA Sequencing facility). Nucleotide sequences for each DLA gene were analyzed and alleles assigned using SBTengine v.3.2 (GenDX, Netherlands). Ambiguous sequences were observed in all dog breeds. In these cases, haplotypes were predicted based on the three-locus haplotypes identified within each of the studied populations. If alleles could not be determined for one or more of the DLA class II genes, the individual was removed from analysis.

### Statistical analysis

Allele frequencies were calculated for each of the three polymorphic DLA genes. Three-locus DLA-DRB1/DQA1/DQB1 haplotypes were determined based on individuals that were homozygous on all three loci (*n* = 63), followed by individuals homozygous at two of the loci (*n* = 87). Odds ratio (OR) estimates were also calculated based on the number of cases and healthy controls carrying a particular haplotype compared to the number of individuals not carrying the haplotype. A 2 × 2 contingency table was used to calculate the ORs and two-tailed Fisher’s exact values for the different haplotypes (http://vassarstats.net/odds2x2.html). The same approach was used to determine statistical differences in homozygosity of the DLA genes between cases and controls [58]. Statistical significance was considered at *p* < 0.05.

Logistic regression was used to model the risk of disease as a function of the observed DLA class II three-locus genotypes. Defining the probability of disease as *p*_*ij*_ for a dog identified with DLA haplotype *i* and DLA haplotype *j*, the logit of this probability was determined as *θ*_*ij*_ =  *log* [*p*_*ij*_/(1 − *p*_*ij*_)]. Modeling the logit as a function of the genotype used the linear model:

$$ {\theta}_{ij}={b}_0+{add}_i+{add}_j+{\mathit{\operatorname{dom}}}_{ij} $$

where *b*_0_ is an unknown constant common to all dogs, *add*_*i*_ is the additive contribution of the *i*-th (*i* = 1,2,3,4,5) haplotype to the risk of disease, *add*_*j*_ is the additive contribution of the *j*-th (*j* = 1,2,3,4,5) haplotype to the risk of disease, and *dom*_*ij*_ is the dominance contribution of both haplotypes *i* and *j* to the risk of disease. Estimation of the unknown additive and dominance effects, along with providing predictions of the risk of disease, were implemented with the Bayesian statistical package Stan [59] executed with the public domain language R [60]. The combination of rare haplotypes and a disease of low prevalence, as seen in the data, increased the risk of empty subclasses. A hierarchical Bayesian model with weakly informative prior distributions for the unknown effects stabilized the estimation process [61].

Specifically, the intercept (*b*_0_) was drawn from the prior density Cauchy (0,10), the additive effects (*add*_*i*_ and *add*_*j*_) were drawn from the prior density N(0,${\sigma}_a^2$), the dominance effects (*dom*_*ij*_) were drawn from the prior density N(0,${\sigma}_d^2$), and the standard deviations of the additive and dominance contributions (*σ*_*a*_ and *σ*_*d*_) were drawn from the positive values of a Cauchy(0,2.5) as recommended for weakly informative priors [62]. The simulation process was conducted across four chains, where each chain was built on a draw of 15,000 total samples, a “burn-in” process of 5000 samples followed by thinning to every 20-th sample. In this way, each chain generated 500 samples, and 2000 samples across the four chains to generate more stable estimates. Convergence of the process was visualized through trace plots of all the unknown values and computation of the Gelman-Rubin statistic for convergence being below 1.05 [63].

## Additional files

Additional file 1: **Table S1.** Allele frequency for the three polymorphic DLA class II genes in 233 bearded collies (DOCX 15 kb)

Additional file 2: **Table S2.** Allele frequency and odds ratio (OR) for Addison’s disease (AD; *n* = 61) vs controls (*n* = 122) in bearded collies. Bolded values were statistically significant at α = 0.05 (DOCX 19 kb)

Additional file 3: **Table S3.** Frequency, code, and odds ratio (OR) with 95% confidence interval (CI) of each three-locus haplotype observed in healthy, Addisonian (AD) and symmetrical lupoid onychodystrophy (SLO) bearded collies in the combined dataset. Text in bold indicates the haplotype frequency significantly differed between cases and controls. Haplotype codes are as used in Table 5; additional codes added as needed. (DOCX 16 kb)

Additional file 4: **Table S4.** Frequency of DLA three-locus haplotypes in European (79 controls, 43 AD and 29 SLO) and North American (80 controls, 45 AD and 19 SLO) bearded collies. Bolded values indicate statistical differences in haplotype frequency according to geographical region as determined by the z-ratio test for independent proportions. Haplotype codes are as used in Table 5; additional codes added as needed. (DOCX 16 kb)

Additional file 5: **Table S5.** Frequency, code, and odds ratio (OR) with 95% confidence interval (CI) of each three-locus haplotype observed in healthy and Addisonian (AD) bearded collies by geographical region. Text in bold indicates the haplotype frequency significantly differed between cases and controls. Haplotype codes are as used in Table 5; additional codes added as needed. (DOCX 17 kb)

Additional file 6: **Table S6.** Frequency of DLA class II alleles segregating in six dog breeds at higher risk for developing Addison’s disease (AD) (DOCX 23 kb)

Additional file 7: **Table S7.** Allele frequency and odds ratio (OR) for Addison’s disease (AD; *n* = 10) vs controls (2n = 10) in West Highland white terriers. Bolded values were statistically significant at α = 0.05 (DOCX 19 kb)

Additional file 8: **Table S8.** Allele frequency and odds ratio (OR) for Addison’s disease (AD; *n* = 11) vs controls (*n* = 13) in Leonbergers. Bolded values were statistically significant at α = 0.05 (DOCX 16 kb)

Additional file 9: **Table S9.** Allele frequency and odds ratio (OR) for Addison’s disease (AD; *n* = 17) vs controls (*n* = 76) in Portuguese water dogs. (DOCX 18 kb)

Additional file 10: **Table S10.** Allele frequency and odds ratio (OR) for Addison’s disease (AD; *n* = 30) vs controls (*n* = 55) in standard poodles. (DOCX 19 kb)

Additional file 11: **Table S11.** Allele frequency and odds ratio (OR) for Addison’s disease (AD; *n* = 12) vs controls (*n* = 9) in Labradoodles. (DOCX 18 kb)

Additional file 12: **Table S12.** Allele frequency and odds ratio (OR) for symmetrical lupoid onychodystrophy (SLO; *n* = 50) vs controls (*n* = 122) in bearded collies. Bolded values were statistically significant at α = 0.05. (DOCX 19 kb)

Additional file 13: **Table S13.** Frequency, code, and odds ratio (OR) with 95% confidence interval (CI) of each three-locus haplotype observed in healthy and symmetrical lupoid onychodystrophy (SLO) bearded collies by geographical region. Text in bold indicates the haplotype frequency significantly differed between cases and controls. Haplotype codes are as used in Table 5; additional codes added as needed. (DOCX 16 kb)

Additional file 14: **Table S14.** Primer sequences used for DLA class II haplotyping (DOCX 13 kb)
