# Supplementary material for: DLA class II risk haplotypes for autoimmune diseases in the bearded collie offer insight to autoimmunity signatures across dog breeds
Source: Canine Genet Epidemiol. 2019 Feb 15;6:2. doi: 10.1186/s40575-019-0070-7 (PMC6376674; doi:10.1186/s40575-019-0070-7)
Supplement: Supplementary file 1 — Table S1. Allele frequency for the three polymorphic DLA class II genes in 233 bearded collies (DOCX 15 kb) [file 40575_2019_70_MOESM1_ESM.docx]

**Supplemental Table 1** Allele frequency for the three polymorphic DLA class II genes in 233 bearded collies

|  | Controls  2*n*=244 | AD  2*n* =122 | SLO  2*n* =100 |
| --- | --- | --- | --- |
| DLA-DRB1 | **%** | **%** | **%** |
| 002:01 | 2.5 | 0.0 | 0.0 |
| 009:01 | 9.8 | 20.5 | 1.0 |
| 015:01 | 28.3 | 35.2 | 6.0 |
| 015:02 | 0.4 | 0.0 | 0.0 |
| 018:01 | 58.6 | 44.3 | 93.0 |
| 023:01 | 0.4 | 0.0 | 0.0 |
|  |  |  |  |
| DLA-DQA1 | **%** | **%** | **%** |
| 001:01 | 68.4 | 64.8 | 94.0 |
| 003:01 | 0.4 | 0.0 | 0.0 |
| 006:01 | 28.7 | 35.2 | 6.0 |
| 009:01 | 2.5 | 0.0 | 0.0 |
|  |  |  |  |
| DLA-DQB1 | **%** | **%** | **%** |
| 001:01 | 2.5 | 0.0 | 0.0 |
| 002:01 | 29.9 | 22.1 | 47.0 |
| 003:01 | 12.7 | 14.8 | 4.0 |
| 005:01 | 0.4 | 0.0 | 0.0 |
| 008:02 | 38.6 | 42.6 | 47.0 |
| 022:01 | 2.0 | 1.6 | 0.0 |
| 023:01 | 13.9 | 18.9 | 2.0 |

*AD* Addison’s disease, *SLO* symmetrical lupoid onychodystrophy
