# Supplementary material for: DLA class II risk haplotypes for autoimmune diseases in the bearded collie offer insight to autoimmunity signatures across dog breeds
Source: Canine Genet Epidemiol. 2019 Feb 15;6:2. doi: 10.1186/s40575-019-0070-7 (PMC6376674; doi:10.1186/s40575-019-0070-7)
Supplement: Supplementary file 2 — Table S2. Allele frequency and odds ratio (OR) for Addison’s disease (AD; n = 61) vs controls (n = 122) in bearded collies. Bolded values were statistically significant at α = 0.05 (DOCX 19 kb) [file 40575_2019_70_MOESM2_ESM.docx]

**Supplemental Table 2** Allele frequency and odds ratio (OR) for Addison’s disease (AD; *n*=61) vs controls (*n*=122) in bearded collies. Bolded values were statistically significant at α=0.05

| BEARDED COLLIES | | | | |  |  |
| --- | --- | --- | --- | --- | --- | --- |
|  | Controls (2*n*=244) | | AD  (2*n*=122) | | OR (95% CI) | p-value^†^ |
| DLA-DRB1 | 2*n* | % | 2*n* | % |  |  |
| 002:01 | 6 | 2.5 | 0 | 0.0 | N/A |  |
| 009:01 | 24 | 9.8 | 25 | 20.5 | **2.36 (1.29 – 4.34)** | **0.00584** |
| 015:01 | 69 | 28.3 | 43 | 35.2 | 1.38 (0.87 – 2.20) | 0.18669 |
| 015:02 | 1 | 0.4 | 0 | 0.0 | N/A |  |
| 018:01 | 143 | 58.6 | 54 | 44.3 | **0.56 (0.36 – 0.87)** | **0.01062** |
| 023:01 | 1 | 0.4 | 0 | 0.0 | N/A |  |
|  |  |  |  |  |  |  |
| DLA-DQA1 |  |  |  |  |  |  |
| 001:01 | 167 | 68.4 | 79 | 64.8 | 0.85 (0.54 – 1.34) | 0.55497 |
| 003:01 | 1 | 0.4 | 0 | 0.0 | N/A |  |
| 006:01 | 70 | 28.7 | 43 | 35.2 | 1.35 (0.85 – 2.15) | 0.23020 |
| 009:01 | 6 | 2.5 | 0 | 0.0 | N/A |  |
|  |  |  |  |  |  |  |
| DLA-DQB1 |  |  |  |  |  |  |
| 001:01 | 6 | 2.5 | 0 | 0.0 | N/A |  |
| 002:01 | 73 | 29.9 | 27 | 22.1 | 0.67 (0.40 – 1.11) | 0.13552 |
| 003:01 | 31 | 12.7 | 18 | 14.8 | 1.19 (0.64 – 2.22) | 0.62631 |
| 005:01 | 1 | 0.4 | 0 | 0.0 | N/A |  |
| 008:02 | 94 | 38.6 | 52 | 42.6 | 1.19 (0.76 – 1.84) | 0.49722 |
| 022:01 | 5 | 2.0 | 2 | 1.6 | 0.80 (0.15 – 4.17) | 1 |
| 023:01 | 34 | 13.9 | 23 | 18.9 | 1.43 (0.80 – 2.56) | 0.28431 |

*N/A* not enough data points to calculate; ^†^Fisher’s exact p-value, significant at p < 0.05
