# Supplementary material for: DLA class II risk haplotypes for autoimmune diseases in the bearded collie offer insight to autoimmunity signatures across dog breeds
Source: Canine Genet Epidemiol. 2019 Feb 15;6:2. doi: 10.1186/s40575-019-0070-7 (PMC6376674; doi:10.1186/s40575-019-0070-7)
Supplement: Supplementary file 4 — Table S4. Frequency of DLA three-locus haplotypes in European (79 controls, 43 AD and 29 SLO) and North American (80 controls, 45 AD and 19 SLO) bearded collies. Bolded values indicate statistical differences in haplotype frequency according to geographical region as determined by the z-ratio test for independent proportions. Haplotype codes are as used in Table 5; additional codes added as needed. (DOCX 16 kb) [file 40575_2019_70_MOESM4_ESM.docx]

**Supplemental Table 4** Frequency of DLA three-locus haplotypes in European (79 controls, 43 AD and 29 SLO) and North American (80 controls, 45 AD and 19 SLO) bearded collies. Bolded values indicate statistical differences in haplotype frequency according to geographical region as determined by the z-ratio test for independent proportions.

|  |  | Controls | | | |  | AD | | | |  | SLO | | | |  |
| --- | --- | --- | --- | --- | --- | --- | --- | --- | --- | --- | --- | --- | --- | --- | --- | --- |
|  | Haplotype | Europe  2*n*=158 | | N. America  2*n*=160 | | p-value | Europe  2*n*=86 | | N. America  2*n*=90 | | p-value | Europe  2*n*=58 | | N. America  2*n*=38 | | p-value |
| code | DLA-DRB1/DQA1/DQB1 | 2n | % | 2n | % |  | 2n | % | 2n | % |  | 2n | % | 2n | % |  |
| 1 | 009:01/001:01/008:02 | 18 | 11.4 | 11 | 6.9 | 0.1618 | 26 | 30.2 | 17 | 18.9 | 0.0799 | 0 | 0.0 | 0 | 0.0 | N/A |
| 2 | 015:01/006:01/003:01 | 13 | 8.2 | 24 | 15.0 | 0.0597 | **6** | **7.0** | **16** | **17.8** | **0.0303** | 2 | 3.4 | 2 | 5.3 | N/A |
| 3 | 015:01/006:01/023:01 | **10** | **6.3** | **29** | **18.1** | **0.0013** | **10** | **11.6** | **21** | **23.3** | **0.0416** | 1 | 1.7 | 1 | 2.6 | N/A |
| 4 | 018:01/001:01/002:01 | **62** | **39.3** | **42** | **26.3** | **0.0135** | 25 | 29.1 | 16 | 17.8 | 0.0766 | 32 | 55.2 | 15 | 39.5 | 0.1323 |
| 5 | 018:01/001:01/008:02 | 45 | 28.5 | 42 | 26.3 | 0.6556 | 16 | 18.6 | 18 | 20.0 | 0.8150 | 23 | 39.7 | 20 | 52.6 | 0.2113 |
| 6 | 015:01/006:01/022:01 | 0 | 0.0 | 5 | 3.1 | N/A | 0 | 0.0 | 2 | 2.2 | N/A | 0 | 0.0 | 0 | 0.0 | N/A |
| 7 | 002:01/009:01/001:01 | 2 | 1.3 | 5 | 3.1 | N/A | 0 | 0.0 | 0 | 0.0 | N/A | 0 | 0.0 | 0 | 0.0 | N/A |
| 8 | 023:01/003:01/005:01 | 1 | 0.6 | 1 | 0.6 | N/A | 0 | 0.0 | 0 | 0.0 | N/A | 0 | 0.0 | 0 | 0.0 | N/A |
| 9 | 015:02/006:01/023:01 | 0 | 0 | 1 | 0.6 | N/A | 1 | 1.2 | 0 | 0.0 | N/A | 0 | 0.0 | 0 | 0.0 | N/A |
| 10 | 006:01/005:01:1/007:01 | 2 | 1.3 | 0 | 0.0 | N/A | 0 | 0.0 | 0 | 0.0 | N/A | 0 | 0.0 | 0 | 0.0 | N/A |
| 11 | 009:01/001:01/008:01:1 | 2 | 1.3 | 0 | 0.0 | N/A | 1 | 1.2 | 0 | 0.0 | N/A | 0 | 0.0 | 0 | 0.0 | N/A |
| 12 | 013:01/001:01/002:01 | 1 | 0.6 | 0 | 0.0 | N/A | 0 | 0.0 | 0 | 0.0 | N/A | 0 | 0.0 | 0 | 0.0 | N/A |
| 13 | 015:01/006:01/020:02 | 1 | 0.6 | 0 | 0.0 | N/A | 0 | 0.0 | 0 | 0.0 | N/A | 0 | 0.0 | 0 | 0.0 | N/A |
| 30 | 003:01/001:01/008:02 | 1 | 0.6 | 0 | 0.0 | N/A | 0 | 0.0 | 0 | 0.0 | N/A | 0 | 0.0 | 0 | 0.0 | N/A |
| 31 | Eg51v/017:01/038:01 | 0 | 0.0 | 0 | 0.0 | N/A | 1 | 1.2 | 0 | 0.0 | N/A | 0 | 0.0 | 0 | 0.0 | N/A |

*N/A* not enough data points to calculate
