# Supplementary material for: DLA class II risk haplotypes for autoimmune diseases in the bearded collie offer insight to autoimmunity signatures across dog breeds
Source: Canine Genet Epidemiol. 2019 Feb 15;6:2. doi: 10.1186/s40575-019-0070-7 (PMC6376674; doi:10.1186/s40575-019-0070-7)
Supplement: Supplementary file 5 — Table S5. Frequency, code, and odds ratio (OR) with 95% confidence interval (CI) of each three-locus haplotype observed in healthy and Addisonian (AD) bearded collies by geographical region. Text in bold indicates the haplotype frequency significantly differed between cases and controls. Haplotype codes are as used in Table 5; additional codes added as needed. (DOCX 17 kb) [file 40575_2019_70_MOESM5_ESM.docx]

**Supplemental Table 5** Frequency, code, and odds ratio (OR) with 95% confidence interval (CI) of each three-locus haplotype observed in healthy and Addisonian (AD) bearded collies by geographical region. Text in bold indicates the haplotype frequency significantly differed between cases and controls. Haplotype codes are as used in Table 5; additional codes added as needed.

|  |  | Europe | | | | | | | North America | | | | | | |
| --- | --- | --- | --- | --- | --- | --- | --- | --- | --- | --- | --- | --- | --- | --- | --- |
|  | Haplotype | Controls | | AD | |  |  |  | Controls | | AD | |  |  |  |
| code | DLA-DRB1/DQA1/DQB1 | 2n | % | 2n | % | OR | 95% CI | p-value^†^ | 2n | % | 2n | % | OR | 95% CI | p-value^†^ |
| 1 | 009:01/001:01/008:02 | 18 | 11.4 | 26 | 30.2 | **3.37** | **1.72-6.60** | **0.0004** | 11 | 6.9 | 17 | 18.9 | **3.15** | **1.41-7.08** | **0.0059** |
| 2 | 015:01/006:01/003:01 | 13 | 8.2 | 6 | 7 | 0.84 | 0.31-2.29 | 0.8073 | 24 | 15.0 | 16 | 17.8 | 1.23 | 0.61-2.45 | 0.5924 |
| 3 | 015:01/006:01/023:01 | 10 | 6.3 | 10 | 11.6 | 1.95 | 0.78-4.88 | 0.2206 | 29 | 18.1 | 21 | 23.3 | 1.37 | 0.73-2.59 | 0.3281 |
| 4 | 018:01/001:01/002:01 | 62 | 39.2 | 25 | 29.1 | 0.63 | 0.36-1.12 | 0.1253 | 42 | 26.3 | 16 | 17.8 | 0.61 | 0.32-1.16 | 0.1601 |
| 5 | 018:01/001:01/008:02 | 45 | 28.5 | 16 | 18.6 | 0.57 | 0.30-1.09 | 0.1212 | 42 | 26.3 | 18 | 20.0 | 0.7 | 0.38-1.31 | 0.2846 |
| 6 | 015:01/006:01/022:01 | 0 | 0 | 0 | 0 | N/A |  |  | 5 | 3.1 | 2 | 2.2 | 0.7 | 0.13-3.71 | 1 |
| 7 | 002:01/009:01/001:01 | 2 | 1.3 | 0 | 0 | N/A |  |  | 5 | 3.1 | 0 | 0.0 | N/A |  |  |
| 8 | 023:01/003:01/005:01 | 1 | 0.6 | 0 | 0 | N/A |  |  | 1 | 0.6 | 0 | 0.0 | N/A |  |  |
| 9 | 015:02/006:01/023:01 | 0 | 0 | 1 | 1.2 | N/A |  |  | 1 | 0.6 | 0 | 0.0 | N/A |  |  |
| 10 | 006:01/005:01:1/007:01 | 2 | 1.3 | 0 | 0 | N/A |  |  | 0 | 0.0 | 0 | 0.0 | N/A |  |  |
| 11 | 009:01/001:01/008:01:1 | 2 | 1.3 | 1 | 1.2 | 0.92 | 0.08-10.27 | 1 | 0 | 0.0 | 0 | 0.0 | N/A |  |  |
| 12 | 013:01/001:01/002:01 | 1 | 0.6 | 0 | 0 | N/A |  |  | 0 | 0.0 | 0 | 0.0 | N/A |  |  |
| 13 | 015:01/006:01/020:02 | 1 | 0.6 | 0 | 0 | N/A |  |  | 0 | 0.0 | 0 | 0.0 | N/A |  |  |
| 30 | 003:01/001:01/008:02 | 1 | 0.6 | 0 | 0 | N/A |  |  | 0 | 0.0 | 0 | 0.0 | N/A |  |  |
| 31 | Eg51v/017:01/038:01 | 0 | 0 | 1 | 1.2 | N/A |  |  | 0 | 0.0 | 0 | 0.0 | N/A |  |  |

*N/A* not enough data points to calculate; ^†^Fisher’s exact p-value, significant at p < 0.05
