# Supplementary material for: DLA class II risk haplotypes for autoimmune diseases in the bearded collie offer insight to autoimmunity signatures across dog breeds
Source: Canine Genet Epidemiol. 2019 Feb 15;6:2. doi: 10.1186/s40575-019-0070-7 (PMC6376674; doi:10.1186/s40575-019-0070-7)
Supplement: Supplementary file 6 — Table S6. Frequency of DLA class II alleles segregating in six dog breeds at higher risk for developing Addison’s disease (AD) (DOCX 23 kb) [file 40575_2019_70_MOESM6_ESM.docx]

**Supplemental Table 6** Frequency of DLA class II alleles segregating in six dog breeds at higher risk for developing Addison’s disease (AD)

|  | Bearded collie | | Labradoodle | | Leonberger | | Portuguese water dog | | Standard poodle | | West Highland white terrier | |
| --- | --- | --- | --- | --- | --- | --- | --- | --- | --- | --- | --- | --- |
|  | **Controls 2*n*=244** | **AD 2*n*=122** | **Controls 2*n*= 18** | **AD 2*n*=24** | **Controls 2*n*=26** | **AD 2*n*=22** | **Controls 2*n*=152** | **AD 2*n*=34** | **Controls 2*n*=110** | **AD 2*n*=60** | **Controls 2*n*=332** | **AD 2*n*=86** |
| DLA-DRB1 | **%** | **%** | **%** | **%** | **%** | **%** | **%** | **%** | **%** | **%** | **%** | **%** |
| 001:01 | - | - | 11.1 | 12.5 | 42.3 | 72.8 | 37.5 | 44.1 | 4.6 | - | 64.2 | 75.6 |
| 002:01 | 2.5 | - | - | 4.2 | - | - | - | - | 1.8 | - | - | - |
| 006:01 | - | - | 5.6 | 8.3 | 19.2 | 4.5 | - | - | 3.6 | - | 0.6 | - |
| 008:02 | - | - | - | - | - | - | 38.9 | 26.5 | - | - | - | - |
| 009:01 | 9.8 | 20.5 | 5.6 | 12.5 | - | - | 5.3 | - | 4.6 | 3.3 | 0.9 | 2.3 |
| 011:01 | - | - | - | - | - | - | 1.3 | - | - | - | - | - |
| 012:01 | - | - | - | - | - | - | 2.0 | - | 3.6 | - | - | - |
| 013:01 | - | - | - | - | 15.4 | 18.2 | - | - | 0.9 | - | - | 1.2 |
| 015:01 | 28.3 | 35.2 | 33.3 | 50.0 | - | - | - | - | 69.1 | 76.7 | 32.5 | 20.9 |
| 015:02 | 0.4 | - | 22.2 | 12.5 | - | - | 7.2 | 14.7 | 6.4 | 15.0 | 1.2 | - |
| 015:03 | - | - | 11.1 | - | - | - | - | - | 3.6 | 3.3 | - | - |
| 016:01 | - | - | - | - | 23.1 | 4.5 | - | - | - | - | - | - |
| 017:01 | - | - | - | - | - | - | - | - | - | - | 0.3 | - |
| 018:01 | 58.6 | 44.3 | - | - | - | - | - | - | - | - | 0.3 | - |
| 020:01 | - | - | - | - | - | - | 0.6 | - | 1.8 | 1.7 | - | - |
| 023:01 | 0.4 | - | - | - | - | - | 7.2 | 14.7 | - | - | - | - |
| 084:01 | - | - | 11.1 | - | - | - | - | - | - | - | - | - |
| DLA-DQA1 | **%** | **%** | **%** | **%** | **%** | **%** | **%** | **%** | **%** | **%** | **%** | **%** |
| 001:01 | 68.4 | 64.8 | 16.7 | 25.0 | 80.8 | 95.5 | 42.8 | 44.1 | 10.0 | 3.3 | 42.2 | 64.0 |
| 002:01 | - | - | - | - | - | - | 1.3 | - | - | - | 0.3 | - |
| 003:01 | 0.4 | - | - | - | - | - | 46.1 | 41.2 | - | - | - | - |
| 004:01 | - | - | - | - | - | - | 2.6 | - | 5.4 | 1.7 | - | - |
| 006:01 | 28.7 | 35.2 | 50.0 | 58.4 | - | - | 7.2 | 14.7 | 71.9 | 80.0 | 29.5 | 12.8 |
| 009:01 | 2.5 | - | 27.7 | 8.3 | - | - | - | - | 9.1 | 15.0 | 27.4 | 23.2 |
| 005:01:1 | - | - | 5.6 | 8.3 | 19.2 | 4.5 | - | - | 3.6 | - | 0.6 | - |
| DLA-DQB1 | **%** | **%** | **%** | **%** | **%** | **%** | **%** | **%** | **%** | **%** | **%** | **%** |
| 001:01 | 2.5 | - | 27.7 | 8.3 | - | - | - | - | 9.1 | 15.0 | 27.4 | 23.2 |
| 002:01 | 29.9 | 22.1 | 11.1 | 8.3 | 80.8 | 95.5 | 37.5 | 44.1 | 3.6 | - | 39.5 | 61.7 |
| 003:01 | 12.7 | 14.8 | - | - | - | - | - | - | - | - | 0.3 | - |
| 004:01 | - | - | - | - | - | - | 38.8 | 26.5 | - | - | - | - |
| 005:01 | 0.4 | - | - | - | - | - | 7.2 | 14.7 | - | - | - | - |
| 007:01 | - | - | 5.6 | 8.3 | 19.2 | 4.5 | - | - | 3.6 | - | 0.6 | - |
| 008:02 | 38.6 | 42.6 | - | - | - | - | - | - | - | - | - | - |
| 011:01 | - | - | - | - | - | - | - | - | - | - | 7.5 | 5.8 |
| 013-017 | - | - | - | - | - | - | - | - | 3.6 | - | - | - |
| 013:03 | - | - | - | - | - | - | 4.0 | - | 1.8 | 1.7 | 0.3 | - |
| 020:02 | - | - | - | - | - | - | - | - | - | - | 15.4 | 7.0 |
| 022:01 | 2.0 | 1.6 | - | 4.2 | - | - | - | - | - | - | - | - |
| 023:01 | 13.9 | 18.9 | 44.4 | 45.9 | - | - | 7.2 | 14.7 | 71.9 | 78.3 | 6.3 | - |
| 026:01 | - | - | 5.6 | 8.3 | - | - | - | - | - | 1.7 | - | - |
| 036:01 | - | - | - | 4.2 | - | - | - | - | 1.8 | - | 1.8 | - |
| 008:01:1 | - | - | 5.6 | 12.5 | - | - | 5.3 | - | 4.6 | 3.3 | 0.9 | 2.3 |

N.B. DLA-DQB1*013-017 is short hand for DQB1*01303 and 01701 appearing on the same haplotype.
