# Supplementary material for: DLA class II risk haplotypes for autoimmune diseases in the bearded collie offer insight to autoimmunity signatures across dog breeds
Source: Canine Genet Epidemiol. 2019 Feb 15;6:2. doi: 10.1186/s40575-019-0070-7 (PMC6376674; doi:10.1186/s40575-019-0070-7)
Supplement: Supplementary file 7 — Table S7. Allele frequency and odds ratio (OR) for Addison’s disease (AD; n = 10) vs controls (2n = 10) in West Highland white terriers. Bolded values were statistically significant at α = 0.05 (DOCX 19 kb) [file 40575_2019_70_MOESM7_ESM.docx]

**Supplemental Table 7** Allele frequency and odds ratio (OR) for Addison’s disease (AD; *n* = 10) vs controls (2*n* = 10) in West Highland white terriers. Bolded values were statistically significant at α=0.05

| WEST HIGHLAND WHITE TERRIERS | | | | |  |  |
| --- | --- | --- | --- | --- | --- | --- |
|  | Controls (2*n*=332) | | AD  (2*n* =86) | | OR (95% CI) | p-value^†^ |
| DLA-DRB1 | 2*n* | % | 2*n* | % |  |  |
| 001:01 | 213 | 64.2 | 65 | 75.6 | 1.73 (1.01 - 2.97) | 0.0542 |
| 006:01 | 2 | 0.6 | 0 | 0 | N/A |  |
| 009:01 | 3 | 0.9 | 2 | 2.3 | 2.61 (0.43 - 15.88) | 0.5882 |
| 013:01 | 0 | 0 | 1 | 1.2 | N/A |  |
| 015:01 | 108 | 32.5 | 18 | 20.9 | **0.55 (0.31 - 0.97)** | **0.0474** |
| 015:02 | 4 | 1.2 | 0 | 0 | N/A |  |
| 017:01 | 1 | 0.3 | 0 | 0 | N/A |  |
| 018:01 | 1 | 0.3 | 0 | 0 | N/A |  |
|  |  |  |  |  |  |  |
| DLA-DQA1 |  |  |  |  |  |  |
| 001:01 | 140 | 42.2 | 55 | 64.0 | **2.43 (1.49 - 3.98)** | **0.0004** |
| 002:01 | 1 | 0.3 | 0 | 0 | N/A |  |
| 006:01 | 98 | 29.5 | 11 | 12.8 | **0.35 (0.18 - 0.69)** | **0.0023** |
| 009:01 | 91 | 27.4 | 20 | 23.2 | 0.80 (0.46 – 1.40) | 0.4946 |
| 005:01:1 | 2 | 0.6 | 0 | 0 | N/A |  |
|  |  |  |  |  |  |  |
| DLA-DQB1 |  |  |  |  |  |  |
| 001:01 | 91 | 27.4 | 20 | 23.2 | 0.80 (0.46 – 1.40) | 0.4946 |
| 002:01 | 131 | 39.5 | 53 | 61.7 | **2.46 (1.51 - 4.01)** | **0.0003** |
| 003:01 | 1 | 0.3 | 0 | 0 | N/A |  |
| 007:01 | 2 | 0.6 | 0 | 0 | N/A |  |
| 011:01 | 25 | 7.5 | 5 | 5.8 | 0.76 (0.28 - 2.04) | 0.6499 |
| 013:03 | 1 | 0.3 | 0 | 0 | N/A |  |
| 020:02 | 51 | 14.5 | 6 | 7.0 | 0.41 (0.17 - 1.00) | 0.0514 |
| 023:01 | 21 | 6.3 | 0 | 0 | N/A |  |
| 036:01 | 6 | 1.8 | 0 | 0 | N/A |  |
| 008:01:1 | 3 | 0.9 | 2 | 2.3 | 2.61 (0.43 - 15.88) | 0.5882 |

*N/A* not enough data points to calculate
