# Supplementary material for: DLA class II risk haplotypes for autoimmune diseases in the bearded collie offer insight to autoimmunity signatures across dog breeds
Source: Canine Genet Epidemiol. 2019 Feb 15;6:2. doi: 10.1186/s40575-019-0070-7 (PMC6376674; doi:10.1186/s40575-019-0070-7)
Supplement: Supplementary file 8 — Table S8. Allele frequency and odds ratio (OR) for Addison’s disease (AD; n = 11) vs controls (n = 13) in Leonbergers. Bolded values were statistically significant at α = 0.05 (DOCX 16 kb) [file 40575_2019_70_MOESM8_ESM.docx]

**Supplemental Table 8** Allele frequency and odds ratio (OR) for Addison’s disease (AD; *n*=11) vs controls (*n*=13) in Leonbergers. Bolded values were statistically significant at α=0.05

| LEONBERGERS |  | | | | |  |
| --- | --- | --- | --- | --- | --- | --- |
|  | Controls  (2*n*=26) | | AD  (2*n*=22) | | OR (95% CI) | p-value^†^ |
| DLA-DRB1 | 2*n* | % | 2*n* | % |  |  |
| 001:01 | 11 | 42.3 | 16 | 72.8 | **3.64 (1.07 - 12.30)** | **0.0447** |
| 006:01 | 5 | 19.2 | 1 | 4.5 | 0.2 (0.02 - 1.86) | 0.1986 |
| 013:01 | 4 | 15.4 | 4 | 18.2 | 1.22 (0.27 - 5.59) | 1 |
| 016:01 | 6 | 23.1 | 1 | 4.5 | 0.16 (0.02 - 1.44) | 0.1064 |
|  |  |  |  |  |  |  |
| DLA-DQA1 |  |  |  |  |  |  |
| 001:01 | 21 | 80.8 | 21 | 95.5 | 5.0 (0.54 - 46.53) | 0.1986 |
| 005:01:1 | 5 | 19.2 | 1 | 4.5 | 0.2 (0.02 - 1.86) | 0.1986 |
|  |  |  |  |  |  |  |
| DLA-DQB1 |  |  |  |  |  |  |
| 002:01 | 21 | 80.8 | 21 | 95.5 | 5.0 (0.54 - 46.53) | 0.1986 |
| 007:01 | 5 | 19.2 | 1 | 4.5 | 0.2 (0.02 - 1.86) | 0.1986 |

^†^Fisher’s exact p-value, significant at p < 0.05
