# Supplementary material for: DLA class II risk haplotypes for autoimmune diseases in the bearded collie offer insight to autoimmunity signatures across dog breeds
Source: Canine Genet Epidemiol. 2019 Feb 15;6:2. doi: 10.1186/s40575-019-0070-7 (PMC6376674; doi:10.1186/s40575-019-0070-7)
Supplement: Supplementary file 9 — Table S9. Allele frequency and odds ratio (OR) for Addison’s disease (AD; n = 17) vs controls (n = 76) in Portuguese water dogs. (DOCX 18 kb) [file 40575_2019_70_MOESM9_ESM.docx]

**Supplemental Table 9** Allele frequency and odds ratio (OR) for Addison’s disease (AD; *n*=17) vs controls (*n*=76) in Portuguese water dogs.

| PORTUGUESE WATER DOGS | | | | |  | |
| --- | --- | --- | --- | --- | --- | --- |
|  | Controls  (2*n*=152) | | AD  (2*n*=34) | | OR (95% CI) | p-value^†^ |
| DLA-DRB1 | 2*n* | % | 2*n* | % |  |  |
| 001:01 | 57 | 37.5 | 15 | 44.1 | 1.32 (0.62 - 2.79) | 0.5597 |
| 008:02 | 59 | 38.9 | 9 | 26.5 | 0.57 (0.25 - 1.30) | 0.2373 |
| 009:01 | 8 | 5.3 | 0 | 0 | N/A |  |
| 011:01 | 2 | 1.3 | 0 | 0 | N/A |  |
| 012:01 | 3 | 2.0 | 0 | 0 | N/A |  |
| 015:02 | 11 | 7.2 | 5 | 14.7 | 2.21 (0.71 - 6.84) | 0.1775 |
| 020:01 | 1 | 0.6 | 0 | 0 | N/A |  |
| 023:01 | 11 | 7.2 | 5 | 14.7 | 2.21 (0.71 - 6.84) | 0.1775 |
|  |  |  |  |  |  |  |
| DLA-DQA1 |  |  |  |  |  |  |
| 001:01 | 65 | 42.8 | 15 | 44.1 | 1.06 (0.50 - 2.24) | 1 |
| 002:01 | 2 | 1.3 | 0 | 0 | N/A |  |
| 003:01 | 70 | 46.1 | 14 | 41.2 | 0.82 (0.39 - 1.74) | 0.7041 |
| 004:01 | 4 | 2.6 | 0 | 0 | N/A |  |
| 006:01 | 11 | 7.2 | 5 | 14.7 | 2.21 (0.71 - 6.84) | 0.1775 |
|  |  |  |  |  |  |  |
| DLA-DQB1 |  |  |  |  |  |  |
| 002:01 | 57 | 37.5 | 15 | 44.1 | 1.32 (0.62 - 2.79) | 0.5597 |
| 004:01 | 59 | 38.8 | 9 | 26.5 | 0.57 (0.25 - 1.30) | 0.2373 |
| 005:01 | 11 | 7.2 | 5 | 14.7 | 2.21 (0.71 - 6.84) | 0.1775 |
| 013:03 | 6 | 4.0 | 0 | 0 | N/A |  |
| 023:01 | 11 | 7.2 | 5 | 14.7 | 2.21 (0.71 - 6.84) | 0.1775 |
| 008:01:1 | 8 | 5.3 | 0 | 0 | N/A |  |

*N/A* not enough data points to calculate

^†^Fisher’s exact p-value, significant at p < 0.05
