# Supplementary material for: DLA class II risk haplotypes for autoimmune diseases in the bearded collie offer insight to autoimmunity signatures across dog breeds
Source: Canine Genet Epidemiol. 2019 Feb 15;6:2. doi: 10.1186/s40575-019-0070-7 (PMC6376674; doi:10.1186/s40575-019-0070-7)
Supplement: Supplementary file 10 — Table S10. Allele frequency and odds ratio (OR) for Addison’s disease (AD; n = 30) vs controls (n = 55) in standard poodles. (DOCX 19 kb) [file 40575_2019_70_MOESM10_ESM.docx]

**Supplemental Table 10** Allele frequency and odds ratio (OR) for Addison’s disease (AD; *n*=30) vs controls (*n*=55) in standard poodles.

| STANDARD POODLES | | | | |  |  |
| --- | --- | --- | --- | --- | --- | --- |
|  | Controls  (2*n*=110) | | AD  (2*n*=60) | | OR (95% CI) | p-value^†^ |
| DLA-DRB1 | 2*n* | % | 2*n* | % |  |  |
| 001:01 | 5 | 4.6 | 0 | 0 | N/A |  |
| 002:01 | 2 | 1.8 | 0 | 0 | N/A |  |
| 006:01 | 4 | 3.6 | 0 | 0 | N/A |  |
| 009:01 | 5 | 4.6 | 2 | 3.3 | 0.72 (0.14 - 3.85) | 1 |
| 012:01 | 4 | 3.6 | 0 | 0 | N/A |  |
| 013:01 | 1 | 0.9 | 0 | 0 | N/A |  |
| 015:01 | 76 | 69.1 | 46 | 76.7 | 1.47 (0.71 - 3.03) | 0.3731 |
| 015:02 | 7 | 6.4 | 9 | 15.0 | 2.60 (0.91 - 7.37) | 0.0967 |
| 015:03 | 4 | 3.6 | 2 | 3.3 | 0.91 (0.16 - 5.14) | 1 |
| 020:01 | 2 | 1.8 | 1 | 1.7 | 0.92 (0.08 - 10.31) | 1 |
|  |  |  |  |  |  |  |
| DLA-DQA1 |  |  |  |  |  |  |
| 001:01 | 11 | 10.0 | 2 | 3.3 | 0.31 (0.07 - 1.45) | 0.1418 |
| 004:01 | 6 | 5.4 | 1 | 1.7 | 0.29 (0.03 - 2.50) | 0.4235 |
| 006:01 | 79 | 71.9 | 48 | 80.0 | 1.57 (0.74 - 3.35) | 0.2722 |
| 009:01 | 10 | 9.1 | 9 | 15.0 | 1.76 (0.67 - 4.62) | 0.3088 |
| 005:01:1 | 4 | 3.6 | 0 | 0 | N/A |  |
|  |  |  |  |  |  |  |
| DLA-DQB1 |  |  |  |  |  |  |
| 001:01 | 10 | 9.1 | 9 | 15.0 | 1.76 (0.67 - 4.62) | 0. 3088 |
| 002:01 | 4 | 3.6 | 0 | 0 | N/A |  |
| 007:01 | 4 | 3.6 | 0 | 0 | N/A |  |
| 013:01:7 | 4 | 3.6 | 0 | 0 | N/A |  |
| 013:03 | 2 | 1.8 | 1 | 1.7 | 0.92 (0.08 - 10.31) | 1 |
| 023:01 | 79 | 71.9 | 47 | 78.3 | 1.42 (0.68 - 2.98) | 0.3684 |
| 026:01 | 0 | 0 | 1 | 1.7 | N/A |  |
| 036:01 | 2 | 1.8 | 0 | 0 | N/A |  |
| 008:01:1 | 5 | 4.6 | 2 | 3.3 | 0.72 (0.14 - 3.85) | 1 |

*N/A* not enough data points to calculate

^†^Fisher’s exact p-value, significant at p < 0.05
