# Supplementary material for: DLA class II risk haplotypes for autoimmune diseases in the bearded collie offer insight to autoimmunity signatures across dog breeds
Source: Canine Genet Epidemiol. 2019 Feb 15;6:2. doi: 10.1186/s40575-019-0070-7 (PMC6376674; doi:10.1186/s40575-019-0070-7)
Supplement: Supplementary file 11 — Table S11. Allele frequency and odds ratio (OR) for Addison’s disease (AD; n = 12) vs controls (n = 9) in Labradoodles. (DOCX 18 kb) [file 40575_2019_70_MOESM11_ESM.docx]

**Supplemental Table 11** Allele frequency and odds ratio (OR) for Addison’s disease (AD; *n*=12) vs controls (*n*=9) in Labradoodles.

| LABRADOODLES | | | | |  |  |
| --- | --- | --- | --- | --- | --- | --- |
|  | Controls  (2*n*=18) | | AD  (2*n*=24) | | OR (95% CI) | p-value^†^ |
| DLA-DRB1 | 2*n* | % | 2*n* | % |  |  |
| 001:01 | 2 | 11.1 | 3 | 12.5 | 1.14 (0.17 - 7.67) | 1 |
| 002:01 | 0 | 0 | 1 | 4.2 | N/A |  |
| 006:01 | 1 | 5.6 | 2 | 8.3 | 1.55 (0.13 - 18.50) | 1 |
| 009:01 | 1 | 5.6 | 3 | 12.5 | 2.43 (0.23 - 25.51) | 0.6227 |
| 015:01 | 6 | 33.3 | 12 | 50.0 | 2.00 (0.56 - 7.09) | 0.3530 |
| 015:02 | 4 | 22.2 | 3 | 12.5 | 0.50 (0.10 - 2.58) | 0.6786 |
| 015:03 | 2 | 11.1 | 0 | 0 | N/A |  |
| 084:01 | 2 | 11.1 | 0 | 0 | N/A |  |
|  |  |  |  |  |  |  |
| DLA-DQA1 |  |  |  |  |  |  |
| 001:01 | 3 | 16.7 | 6 | 25.0 | 1.67 (0.36 - 7.82) | 0.7083 |
| 006:01 | 9 | 50.0 | 14 | 58.4 | 1.4 (0.41 - 4.79) | 0.7555 |
| 009:01 | 5 | 27.7 | 2 | 8.3 | 0.24 (0.04 - 1.40) | 0.2080 |
| 005:01:1 | 1 | 5.6 | 2 | 8.3 | 1.55 (0.13 - 18.50) | 1 |
|  |  |  |  |  |  |  |
| DLA-DQB1 |  |  |  |  |  |  |
| 001:01 | 5 | 27.7 | 2 | 8.3 | 0.24 (0.04 - 1.40) | 0.2080 |
| 002:01 | 2 | 11.1 | 2 | 8.3 | 0.73 (0.09 - 5.72) | 1 |
| 007:01 | 1 | 5.6 | 2 | 8.3 | 1.55 (0.13 - 18.50) | 1 |
| 022:01 | 0 | 0 | 1 | 4.2 | N/A |  |
| 023:01 | 8 | 44.4 | 11 | 45.9 | 1.06 (0.31 - 3.61) | 1 |
| 026:01 | 1 | 5.6 | 2 | 8.3 | 1.55 (0.13 - 18.50) | 1 |
| 036:01 | 0 | 0 | 1 | 4.2 | N/A |  |
| 008:01:1 | 1 | 5.6 | 3 | 12.5 | 2.43 (0.23 - 25.51) | 0.6227 |

*N/A* not enough data points to calculate

^†^Fisher’s exact p-value, significant at p < 0.05
