# Supplementary material for: DLA class II risk haplotypes for autoimmune diseases in the bearded collie offer insight to autoimmunity signatures across dog breeds
Source: Canine Genet Epidemiol. 2019 Feb 15;6:2. doi: 10.1186/s40575-019-0070-7 (PMC6376674; doi:10.1186/s40575-019-0070-7)
Supplement: Supplementary file 12 — Table S12. Allele frequency and odds ratio (OR) for symmetrical lupoid onychodystrophy (SLO; n = 50) vs controls (n = 122) in bearded collies. Bolded values were statistically significant at α = 0.05. (DOCX 19 kb) [file 40575_2019_70_MOESM12_ESM.docx]

**Supplemental Table 12** Allele frequency and odds ratio (OR) for symmetrical lupoid onychodystrophy (SLO; *n*=50) vs controls (*n*=122) in bearded collies. Bolded values were statistically significant at α=0.05.

| BEARDED COLLIES | | | | |  |  |
| --- | --- | --- | --- | --- | --- | --- |
|  | Controls (2*n*=244) | | SLO  (2*n*=100) | | OR (95% CI) | p-value^†^ |
| DLA-DRB1 | 2*n* | % | 2*n* | % |  |  |
| 002:01 | 6 | 2.5 | 0 | 0.0 | N/A |  |
| 009:01 | 24 | 9.8 | 1 | 1.0 | **0.09 (0.01 – 0.69)** | **0.00474** |
| 015:01 | 69 | 28.3 | 6 | 6.0 | **0.16 (0.07 – 0.39)** | **6.92 x 10 ^-6^** |
| 015:02 | 1 | 0.4 | 0 | 0.0 | N/A |  |
| 018:01 | 143 | 58.6 | 93 | 93.0 | **9.38 (4.18 – 21.08)** | **7.40 x 10^-10^** |
| 023:01 | 1 | 0.4 | 0 | 0.0 | N/A |  |
|  |  |  |  |  |  |  |
| DLA-DQA1 |  |  |  |  |  |  |
| 001:01 | 167 | 68.4 | 94 | 94.0 | **7.22 (3.03 – 17.21)** | **4.19 x 10 ^-7^** |
| 003:01 | 1 | 0.4 | 0 | 0.0 | N/A |  |
| 006:01 | 70 | 28.7 | 6 | 6.0 | **0.16 (0.07 – 0.38)** | **3.29 x 10 ^-6^** |
| 009:01 | 6 | 2.5 | 0 | 0.0 | N/A |  |
|  |  |  |  |  |  |  |
| DLA-DQB1 |  |  |  |  |  |  |
| 001:01 | 6 | 2.5 | 0 | 0.0 | N/A |  |
| 002:01 | 73 | 29.9 | 47 | 47.0 | **2.08 (1.29 – 3.35)** | **0.00289** |
| 003:01 | 31 | 12.7 | 4 | 4.0 | **0.29 (0.10 – 0.83)** | **0.01721** |
| 005:01 | 1 | 0.4 | 0 | 0.0 | N/A |  |
| 008:02 | 94 | 38.6 | 47 | 47.0 | 1.42 (0.88 – 2.26) | 0.14992 |
| 022:01 | 5 | 2.0 | 0 | 0.0 | N/A |  |
| 023:01 | 34 | 13.9 | 2 | 2.0 | **0.13 (0.03 – 0.54)** | **0.00167** |

*N/A* not enough data points to calculate; ^†^Fisher’s exact p-value, significant at p < 0.05
