# Supplementary material for: DLA class II risk haplotypes for autoimmune diseases in the bearded collie offer insight to autoimmunity signatures across dog breeds
Source: Canine Genet Epidemiol. 2019 Feb 15;6:2. doi: 10.1186/s40575-019-0070-7 (PMC6376674; doi:10.1186/s40575-019-0070-7)
Supplement: Supplementary file 14 — Table S14. Primer sequences used for DLA class II haplotyping (DOCX 13 kb) [file 40575_2019_70_MOESM14_ESM.docx]

**Supplemental Table 14** Primer sequences used for DLA class II haplotyping

| **Primer Name** | **Primer Sequence** | **UCSC Amplicon Location** |
| --- | --- | --- |
| f-DRBIn1 * | CCGTCCCCACAGCACATTTC | chr12:2157050-2157371 |
| r-DRBIn2-T7 * | TAATACGACTCACTATAGGGTGTGTCACACACCTCAGCACCA |  |
| f-DQAIn1 * | TAAGGTTCTTTTCTCCCTCT | chr12:2225052-2225397 |
| r-DQAIn2 * | GGACAGATTCAGTGAAGAGA |  |
| f-DQB1B-T7 * | TAATACGACTCACTATAGGGCTCACTGGCCCGGCTGTCTC | chr12:2248882-2249178 |
| r-DQBR2 * | CACCTCGCCGCTGCAACGTG |  |
| f-DQB1 | CCTGGGAGAGAGGGTGCT | chr12:2248641-2249313 |
| r-DQB1 | AGCAAGCAAGGGGTCTCAG |  |
| f-DRB1 | GTGCTGGTGGTTGGGGTG | chr12:2156855-2157454 |
| r-DRB1 | CACACCCATTCTCTGTCCCC |  |

* Primers described in Bexfield et al., 2012. Underlined sequence corresponds to a T7 tail added to DLA-DRB1 and -DQB1 primers
